# “I would have said ‘bad news’ but now I say ‘different news”: Mixed methods evaluation of a communication skills training for healthcare professionals in the first 1000 days of life

**DOI:** 10.1371/journal.pone.0319092

**Published:** 2025-05-07

**Authors:** Esther Mugweni, Tamsyn Eida, Tracy Pellat-Higgins, Sabrena Jaswal, Melita Madden, Angela Emrys-Jones, Sally Kendall

**Affiliations:** 1 Department of Public Health, Policy and Systems, University of Liverpool, Institute of Population Health, Liverpool, United Kingdom; 2 Public Health Wales, 2 Capital Quarter, Cardiff, United Kingdom; 3 University of Kent, Centre for Health Service Research, Canterbury, Kent, England; 4 Government of Nova Scotia, Department of Labour, Skills and Immigration, Skills and Learning Branch, Floor, Maritime Centre, Halifax, Nova Scotia, Canada; 5 Touchstone, Leeds, West Yorkshire, United Kingdom; 6 National Down Syndrome Policy Group, Tunbridge Wells, England; Murcia University, Spain, SPAIN

## Abstract

**Background:**

Receiving a diagnosis of congenital anomalies in the first 1000 days of life can have significant implications for a family’s emotional and mental wellbeing. We refer to this as different news. We evaluated a communications skills training to improve how healthcare professionals deliver different news using a train-the-trainer (Champions) model.

**Methods:**

We recruited 22 healthcare professionals from 6 NHS trusts in England and trained them as Champions. They delivered 17 training sessions to healthcare professional colleagues. Data were collected on knowledge, skills and attitudes to different news communication using a bespoke questionnaire and the Self-Efficacy Scale (SE-12) at pre-training, straight after training and four weeks post-training. We conducted 19 interviews with healthcare professionals, four managers and eight parents. Data were analysed using Framework analysis guided by the Theoretical Domains Framework.

**Results:**

A total of 204 healthcare professionals completed pre-training questionnaires, 187 completed post-training questionnaires immediately after training, and 109 completed the questionnaires four weeks post-training. A total of 179 healthcare professionals completed the SE-12 scale immediately after training and 102 completed it at four weeks follow-up. The training improved healthcare professionals’ confidence and skills to deliver different news. There were statistically significant differences in confidence levels between pre-/post-training SE-12 scores in delivering different news. Scores were significantly higher post-training. The estimated difference in mean scores post-training was 18.3 (95% confidence interval 15.7–20.9 p < 0.001), and one-month post-training 16.9 (95% confidence interval 13.7–20.2; p < 0.001) more than three times larger than the difference in SE-12 in the validation sample. There was a statistically significant difference between SE-12 scores for the Champions and the healthcare professionals they trained. SE-12 scores were higher for Champions and their improvement from pre-training was greater. Overall, participants reported that the training provided the skills to structure different news conversations, use the right language and pace the provision of information and support.

**Conclusions:**

The results suggest that the training equips healthcare professionals to deliver different news to families sensitively and compassionately which can potentially prevent mental ill-health across the life course.

## Introduction

The first 1000 days covering conception, through pregnancy, to the time a child is two years old, are important for their physical, emotional, and cognitive development [1,2]. This is also a period of increased vulnerability for families particularly if their expected parenting journey is altered by the identification of congenital anomalies (congenital variations.^1^). In England, congenital variations may be identified during pregnancy as part of the NHS Foetal Anomaly Screening Programme, at birth or during various stages of the child’s life through developmental assessments and or diagnostic tests [3,4].

The diagnostic process is pivotal for families, with significant implications for their emotional and mental well-being [4–7]. In this study, we refer to this process as delivering different news (DDN), which is the process of informing parents about their child being diagnosed with a congenital variation in the first 1000 days of life. In previous research, parents experienced various emotions after diagnosis including distress, shock, fear, depression, anxiety and chronic stress [8–13]. Research also shows that positive experiences of receiving different news are associated with greater levels of acceptance of the child’s condition, lower levels of stress, more effective coping strategies and more cooperation with healthcare professionals [5,14].

Ineffective communication can adversely affect parents, leaving them feeling anxious, uncertain and generally dissatisfied with their care [5,6,15]. This is of particular importance in the first 1000 days of a child’s life as parent experiences of chronic stress, depression, anxiety and/or other mental health conditions may affect foetal programming and the parent-foetus relationship – and post-birth, this may affect the parent-infant relationship [16]. This, in turn, can impact the social-emotional, cognitive and physical development of children, increasing their risk of mental illness in later life [17–20]. Perinatal mental health problems cost the NHS approximately £8.1 billion per birth cohort, with 72% of costs being associated with the adverse impact on their child [21]. This data likely presents a very conservative estimate of the economic and human impact of perinatal mental health and further work using longitudinal cohort or big data to better understand the long-term impact on both children and parents is warranted.

Every family that receives different news in the first 1000 days of a child’s life should have access to a healthcare professional who has the knowledge and skills to be able to deliver the news, and subsequent support, sensitively, and compassionately. Currently, not every family has access to such a healthcare professional due to a lack of standardised training or policy to guide professionals on how to effectively deliver different news [4,5,7]. To this end, there is significant variation in the way that different news is delivered by healthcare professionals.

Providing evidenced-based communication skills training to healthcare professionals could potentially minimise the negative psychological impact of the news, maximise the psychological well-being of the whole family and reduce staff burnout [22–24]. Findings from a systematic review have indicated that communication skills training can improve healthcare professionals’ self-efficacy concerning communication tasks including improved confidence to successfully deliver different news [24]. It has also been associated with improvement in healthcare professionals’ ability to show empathy to families as well as increased families’ satisfaction with services [22,23]. Given that most family complaints are related to poor communication as well as an inability to demonstrate care, communication skills training could benefit healthcare professionals, employers and families. Providing healthcare professionals with communication skills training resonates with the cross-government agenda to intervene more actively in the first 1000 days of life to reduce inequality and improve children’s health, development and life chances [2,25].

### Context of the study training

We conducted a feasibility and acceptability study of communication skills training for healthcare professionals involved in the prenatal and postnatal delivery of different news in 2017 which is described elsewhere [5,6,26]. We used the Medical Research Council’s guidelines for the development and evaluation of complex interventions to co-create the training intervention with parents, carers and healthcare professionals [27]. These guidelines suggest that the intervention development be underpinned by a robust theory. To this end, the training we developed made use of the Theoretical Domains Framework [28]. This framework has been used extensively for understanding and overcoming barriers and facilitators to the implementation of new clinical practice or guidelines in the context in which it occurs [28].

**Fig 1:**
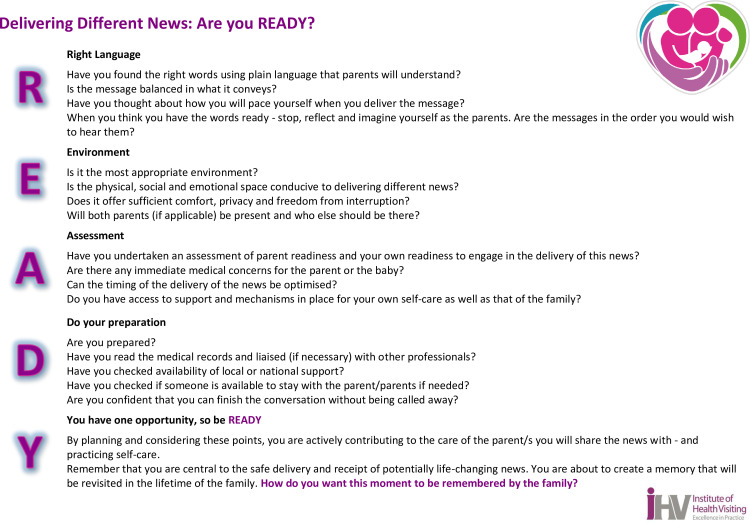
Ready framework.

The training we developed aimed to equip healthcare professionals to demonstrate empathy; show compassion; plan around the demands of their work, utilise kind, simple and truthful language; offer sufficient time to answer questions; and understand how to refer families for further care and support using a structured READY framework (see Fig 1) [5,6]. These key aspects of delivering different news have also been highlighted in other studies as crucial for minimising the negative impact of receiving different news on parents [8,11,15,29]. Due to the small sample size for the feasibility study, it was necessary to conduct a follow-up study to test the findings of the exploratory study with a larger and more diverse group of professionals involved in the first 1000 days of life. This study had the following objectives:

- To update the delivering different news training intervention based on the recommendations from the feasibility study.
- To conduct a pre and post-test study to measure the impact of the training on the self-efficacy, knowledge, attitudes and practices of healthcare professionals who receive the delivering different news training.
- To conduct an in-depth qualitative exploration of the barriers and facilitators to the implementation of the training in practice from the perspective of healthcare professionals and managers.
- To assess the sustainability and scalability of providing the delivering different news training through a cascade model of training.

## Methods

Ethical approval to conduct the Delivering Different News Study 2 (DDN Study 2) was received from the Health Research Authority (REC reference 20/NW/0178). This manuscript adhered to the guidelines for Good Reporting of A Mixed Methods Study (GRAMMS)[30]. The qualitative aspect of the study adhered to the standards for reporting qualitative research guidelines for reporting qualitative studies [31]. The study was conducted in three phases which are described below:

### Phase 1: Updating of the delivering different news training

Phase 1 used a phenomenological approach, as this enables researchers to understand lived experiences [32]. We used purposive sampling to recruit parents with the lived experience of receiving different news about medical conditions which were not captured in the case studies for the training delivered during the feasibility study [33]. This was a key recommendation from that study [5]. Interviews were conducted by EM and SJ who are both experienced health services researchers. The interview guide explored the parents’ experience of receiving different news, what went well and what could have gone better, and their perceptions of training needs for healthcare professionals. All interviews were digitally audio-recorded and analysed by EM and SJ using Framework Analysis guided by the Theoretical Domains Framework [28,34,35]. Other members of the research team supported the data analysis, questioning and interrogating the interpretations of the findings. Framework analysis begins with familiarisation with the data, followed by the development of a thematic framework that is used for indexing the data [35]. This was followed by charting the data and then mapping and interpretation to develop descriptive and explanatory findings [35]. Summaries of the core lived experience discussed in the interviews were used to build case studies for discussion in the updated delivering different news training.

### Phase 2: Training of healthcare professionals

We used a train-the-trainer (cascade model) to deliver the delivering different news training. We worked with the Clinical Research Network to recruit 6 NHS trusts into the study. Each trust identified at least three healthcare professionals with the clinical credibility, professional experience, and confidence to deliver the training to their colleagues and thus become delivering different news Champions. We conducted a one-day training with these healthcare professionals to enable them to understand how to deliver different news and their role as delivering different news Champions which included:

- Training their colleagues and increasing awareness and knowledge of delivering different news within teams, enabling them to support families and get things right when they deliver different news using the READY framework to structure the process of delivering different news.
- Ensuring service users and those who are experts by experience were partners in the planning and shaping of local services.

The training used didactic and experiential learning methodologies including an overview of the delivering different news feasibility study, an oral presentation by a parent with lived experience and a discussion of case studies (informed by the parent interviews from phase 1 and the feasibility study) [5]. The delivering different news Champions were provided with an online set of training materials, trainer resources and guidance and support from the research team to organise, plan and cascade the training. We asked each delivering different news Champion to cascade half-day training to an additional 15 healthcare professionals with support from their managers or principal investigators. Delivery of the training varied according to their trust, their role, and the professional groups of healthcare professionals they were targeting. The training itself followed the same structure as the delivering different news Champion’s training without the section on cascading the training.

### Phase 3: Mixed methods evaluation of the training

#### Quantitative data collection and analysis.

Quantitative data were collected online, using Qualtrics surveys completed at three time points: pre-training, straight after training and four weeks post-training. The survey assessed participants’ knowledge, skills and attitudes towards delivering different news using a bespoke questionnaire as well as the Self-Efficacy Questionnaire (SE-12) for communication. The SE-12 is a validated tool for measuring how healthcare professionals demonstrate empathy skills and relationship-building skills [36]. Self-efficacy is an individual’s confidence in their ability to successfully accomplish a specific task. The concept is drawn from Bandura’s Social Learning Theory [37]. The SE-12 tool has been used by other researchers to evaluate communication skills training. The pretraining survey also contained demographic questions. Each participant was allocated a 4-digit code to use in each survey to enable linking of their pre and post-training data.

#### Quantitative data analysis.

*Primary and secondary outcome measures:* Our primary outcome measure was a change in the healthcare professional scores on the Self-Efficacy Questionnaire (SE-12) [36]. Our secondary outcome measures were changes in the individual items on knowledge, skills and attitudes concerning delivering different news as captured in a bespoke questionnaire.

#### Sample size.

The minimum sample size required to attain 90% statistical power to detect a difference of 5 points or greater in the total SE-12 score between pre- and post-training, assuming a standard deviation of 20 [36] and a significance level of 0.05 (95% confidence interval), was 171 healthcare professionals. The SE-12 difference of 5 points was considered the minimum important change based on the difference between those who had received communications training and those who had not in the validation sample [36]. The Covid-19 pandemic impacted recruitment due to staff absences and increased pressures on services. As such we extended the study by an additional 6 months and managed to recruit 204 healthcare professionals to participate in the training (participant descriptions are in the results section).

#### Analysis process.

Stata/IC software version 16.1 was used to analyse the quantitative data. There was no evidence of systematic occurrences of missing data observed in the pre- and post-training data. Missing values were not estimated, so only observed data were analysed. SE-12 data were analysed using analysis of variance (ANOVA) to compare the mean response pre-and post-training, comparing differences within the participant. The model included fixed effects for the time, site (NHS trust) and Champion (delivering different news Champion or not). The differences between pre- and post-training SE-12 were estimated with 95% confidence intervals. Diagnostic tests and plots to assess the assumptions of normality were performed before analysis. There was some evidence of non-normality for SE-12, a sensitivity non-parametric analysis using the Wilcoxon signed-rank test was performed in addition to the parametric analysis, and medians and interquartile range were presented in the plots.

Individual items from the pre-and post-training questionnaires were analysed using the non-parametric Wilcoxon signed-rank test. Stepwise regression analysis was performed to model the relationship between demographic/professional factors and observed outcomes post-training for SE-12. A significance level of 0.1 was used to determine which factors were added and removed from the regression model. Factors included in this analysis were baseline SE-12, age, gender, profession, language spoken at home, length of employment in current position, prior training in delivering different news (Yes/No), prior training in communication skills (Yes/No), and trainer (Champion or training team ID number or DDN Champion). Summary statistics (mean, SD, median and interquartile range) were calculated and presented in tables.

#### Qualitative data collection and analysis.

We used a phenomenological approach, to help us contextualise the quantitative results by exploring the experience of delivering different news after training [32]. We used purposive sampling to recruit healthcare professionals who had taken part in the delivering different news training. Interviews were conducted by TE, an experienced health services researcher. These explored how the training and READY framework were utilised in the delivery of different news; the perceived changes in knowledge, attitude and practices after the training, the barriers and facilitators to transfer of knowledge into daily practice, and the strengths and limitations of the training. Healthcare professionals were able to register their interest to take part in interviews via the online post-training survey. Interviews with four managers from different NHS trusts were also conducted by the same researcher, to explore the barriers and facilitators to the adoption of the training in their trusts as well as the sustainability of the training. Interviews lasted between 25 and 45 minutes and were conducted virtually due to Covid-19 social-distancing measures. All interviews were digitally audio-recorded and then transcribed.

#### Qualitative data analysis.

All transcripts were organised using NVivo v12 and analysed by two experienced health services researchers (EM and TE) using Framework Analysis guided by the TDF to conduct the analysis. The process of framework analysis has been described in earlier sections this was also used for this phase of the study. The descriptive and explanatory findings were illustrated using various anonymised quotations [35]. Other members of the research team supported the data analysis, questioning and interrogating the interpretations of the findings. We triangulated data from healthcare professionals, seven parent interviews and the quantitative data.

## Results

### Description of quantitative study participants

Between November 2020 and February 2021, we trained 22 delivering different news Champions from six NHS organisations across England through two separate virtual, interactive training days. Our delivering different news Champions were from neonatal medicine, foetal medicine, health visiting, ultrasound and obstetrics. Between February and July 2021, the delivering different news Champions delivered 17 half-day training sessions. Pre-training data were collected for 204 healthcare professionals (Champions and cascade trainees) who participated in the delivering different news training. 187 healthcare professionals completed the bespoke questionnaire straight after training and 109 healthcare professionals at four weeks post training. 179 healthcare professionals completed the SE-12 scale immediately after training and 102 healthcare professionals completed the SE-12 scale at 4 four weeks. The mean age of participants was 44.8 years (SD 9.96), and most participants were female n = 190 (94.1%). Table 1 summarises the characteristics of the study participants.

**Table 1:** Quantitative participant characteristics.

Age (years)		Mean	44.8
	SD	9.96
	Median	44
	25%ile	37.5
	75%ile	52.5
	N	204
Gender	Females	N (%)	190 (94.1)
	Males	N (%)	12 (5.9)
DDN Champion Profession		N (%)	20 (9.8)
	Health Visitor	N (%)	47 (22.9)
	Midwife (including screening midwife)	N (%)	28 (13.7)
	Neonatal Doctor	N (%)	10 (4.9)
	Nursery Nurse	N (%)	15 (7.3)
	Obstetrician or Obstetric Specialty Trainee	N (%)	2 (1.0)
	Paediatric Doctor/Trainee	N (%)	11 (5.4)
	Public Health Nurse	N (%)	7 (3.4)
	Sonographer	N (%)	52 (25.4)
	Specialist paediatric nurses OR Advanced Nurse Practitioners and/or Neonatal nurses	N (%)	21 (10.2)
	Other (please specify)	N (%)	12 (5.8)
Length of time employed in current position	Less than ½ year	N (%)	18 (8.8)
	½ year - less than 1 year	N (%)	16 (7.8)
	1 year – less than 2 years	N (%)	14 (6.8)
	2 years – less than 5 years	N (%)	33 (16.1)
	5 years – less than 10 years	N (%)	53 (25.9)
	More than 10 years	N (%)	71 (34.6)
Language spoken at home	English	N (%)	180 (88.2)
	Other	N (%)	24 (11.8)
Prior training in delivering different news to patients?	Yes	N (%)	40 (19.5)
	No	N (%)	165 (80.5)
Prior participation in communication skills training?	Yes	N (%)	117 (60.6)
	No	N (%)	76 (39.4)

### Description of Qualitative Study Participants

We conducted 19 interviews with healthcare professionals and 4 managers. They comprised of 4 health visitors, 4 sonographers, 4 midwives, a paediatrician, 2 obstetricians, 2 neonatologists, 2 neonatal nurses, and 4 managers (some managers also held clinical roles; 2 attended the training). We also interviewed seven parents as part of phase 1 (updating the training) process and one parent after the training was delivered. Due to the small sample size involved in the interviews, we have omitted the detailed description of participants to protect their anonymity and to ensure confidentiality. We labelled the healthcare professionals by numbers.

## Study Findings

Our findings are categorised according to the relevant TDF domains that emerged from the qualitative data and integrated with the quantitative findings.

### Beliefs about the consequences of the training, skills and knowledge

We examined how the training impacted self-efficacy, knowledge and skills and how the content of the training could lead to expected changes in clinical practice.

#### Confidence.

We measured participants’ self-efficacy (confidence to complete the task of delivering different news) before and after the training using the SE-12. There were statistically significant differences between pre- and post-training SE-12 scores at both post-training time points, which were more than three times larger than the minimum important difference of 5 points observed in the original SE-12 validation study. SE-12 was significantly higher post-training. These comparisons were also significant using the non-parametric Wilcoxon signed rank test as shown in Table 2 below:

**Table 2:** ANOVA estimates of pre-and post-training differences.

	Outcome	Mean post	Mean Pre	Difference	95% CI	ANOVA P-value	Wilcoxon signed rank P-value
Post-training	SE-12	103.9	85.6	18.3	15.7 – 20.9	*<0.001*	*<0.001*
One month	SE-12	102.5	85.6	16.9	13.7 – 20.2	*<0.001*	*<0.001*

These changes were also reflected in the qualitative data. Interviewees described the impact of the training on their confidence:

*“I think the training has helped… however they react you know that you’ve got that knowledge base to act accordingly…. I don’t fear it as much … I would never look forward to it, but I think, I think if anybody has to give it, I want to be one of the people that give it because I’d like to think that I have the knowledge base and the skills to give that news in the best way possible.”* (Healthcare Professional 3 [HCP3])

“I*t definitely made me feel that the next time I’m in that situation, I’m more skilled in how to go about dealing with that and more confident in the phrases and terminology.*” (HCP18)

There were no statistically significant differences in the change from baseline in SE-12 scores between the six trusts, although overall SE-12 scores for Site E were higher compared to the other trusts. This is an important finding which suggests that self-efficacy to deliver different news increased across the board although there was some variation in the changes across the sites.

**Fig 2:**
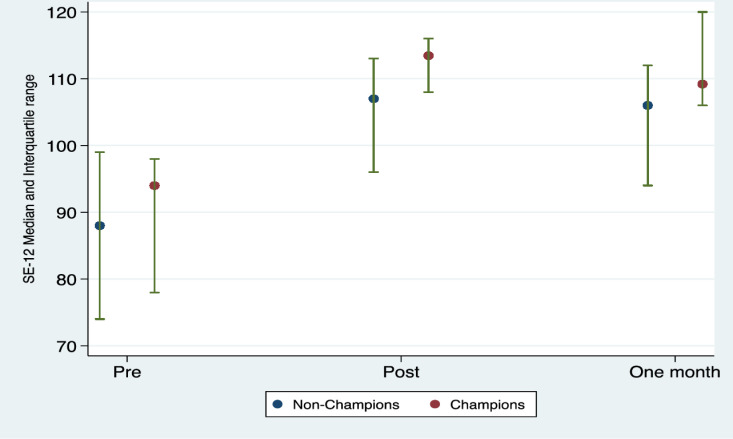
SE-12 Median and Interquartile range over time by Champion.

We examined the data to see if there was a difference in scores between healthcare professionals who were trained by the research team (delivering different news Champions) and those trained by the Champions (non-Champions). There was a statistically significant difference between delivering different news Champions and non-Champions, SE-12 scores were generally higher for Champions and their improvement from pre-training was greater. The difference from non-Champions in SE-12 change scores post-training was 7.81 (95% confidence interval 1.37–14.2) and 13.0 (95% confidence interval 3.10–22.9) one-month post-training (see Fig 2 for details), a greater change than the minimum important difference of 5 points which was in the validated sample [36].

#### Knowledge and skills.

Both the qualitative and the quantitative data indicated that the training had an impact on the self-reported knowledge and skills of the participants. There was a statistically significant increase in the perception that healthcare professionals were confident to deliver different news and that they had the skills to deliver different news after the training as shown in Table 3 below:

**Table 3:** Pre and post-training scores on confidence and skills to deliver different news.

I feel confident in delivering different news to expectant/new parents	Time		
	Pre-training n (%)	Post-training n (%)	One month post-training n (%)
Strongly Disagree	7 (3.7)	2 (1.1)	1 (0.9)
Disagree	41 (21.9)	2 (1.1)	1 (0.9)
Neither Agree nor Disagree	76 (40.6)	20 (10.7)	11 (10.1)
Agree	58 (31.0)	111 (59.4)	70 (64.2)
Strongly Agree	5 (2.7)	52 (27.8)	26 (23.9)
Total	187	187	109
Wilcoxon signed rank test		*<0.001*	*<0.001*
**I have the skills to deliver different news**	Time		
	Pre-training n (%)	Post-training n (%)	One month post-training n (%)
Strongly Disagree	2 (1.1)	1 (0.5)	1 (0.9)
Disagree	22 (11.8)	1 (0.5)	0 (0.0)
Neither Agree nor Disagree	89 (47.6)	13 (7.0)	4 (3.7)
Agree	69 (36.9)	111 (59.4)	79 (72.5)
Strongly Agree	5 (2.7)	61 (32.6)	25 (22.9)
Total	187	187	109
Wilcoxon signed rank test		*<0.001*	*<0.001*

Among other things, the training gave healthcare professionals confidence and skills in the following areas: pacing the provision of information to the needs of the family, the use of the right language when delivering different news and provision of a structure for delivering the different news conversation using the READY mnemonic. We discuss these in detail below:

*Pacing the provision of information:* Healthcare professionals indicated that the training allowed them to rethink how families managed information when experiencing shock and the need to give them time to process the information and access support. This is an important aspect of delivering different news which was also highlighted by parents when we developed the training intervention:

*“I needed 24 hours to come to grips with it and I needed somebody to either take the children or give me 24 hours just to get my head around it because it was such a bombshell...It wipes out everything that you never knew that you were thinking about….”* (Co-Production Parent 1)

*“So the only suggestion that I would make is to give the parents a couple of weeks or so to process the information…..then follow it up with a phone call or email the parent and say, when is a good time for me to give you a follow-up phone call and then give them all the resources but to give it on the day it’s too much...because I misplaced half of what they gave me, I couldn’t remember what’s what, it was so overwhelming.”* (Co-Production Parent 6)

After the training, some healthcare professionals felt better able to give parents opportunities for follow-up and optimise the timing of information sharing to ensure that parents had adequate support:

*“On Friday at half-past four, I got a CVS result that showed a baby had Edward’s syndrome. And I, you know, I just thought, no, I didn’t give that result, you know, my colleagues gave it this morning (Monday) because that lady, you know, she did not need to know that at half-past four on a Friday and then be left with nothing, nowhere to go over the weekend, no one to talk to.” (HPC 3)*

This is an important change to clinical practice which was also highlighted by parents during the co-production process with the need to not leave parents hanging with no one to speak to being emphasised:

*“The way we found out is we just got a letter through from the geneticist saying: “Your daughter has a genetic diagnosis; here’s an appointment time” and it was like three or four weeks away.”* (Co-production Parent 3)

Our quantitative data as shown in Table 4 below, also indicated that healthcare professionals generally felt that they were better able to support parents after the training as shown in the results below which were statistically significant immediately after the training as well as one month after the training:

**Table 4:** Pre and post-training scores on knowledge to provide meaningful support to parents.

I know how to provide parents with meaningful support	Time		
	Pre-training n (%)	Post-training n (%)	One month post-training n (%)
Strongly Disagree	1 (0.5)	2 (1.1)	1 (0.9)
Disagree	17 (9.1)	1 (0.5)	1 (0.9)
Neither Agree nor Disagree	56 (29.9)	14 (7.5)	3 (2.8)
Agree	102 (54.5)	105 (56.1)	69 (63.3)
Strongly Agree	11 (5.9)	65 (34.8)	35 (32.1)
Total	187	187	109
Wilcoxon signed rank test		*<0.001*	*<0.001*

*Right language:* Interviewees described a greater commitment to getting the language right for parents around diagnoses and testing, referring to ‘chance’ rather than risk and being more mindful of their language:

“*Listening to the parent contribute {to the delivering different news training}, listening to them very, kind of clearly repeat what they’d been told even though it’s many years down the line and you know how jarring they found certain phrases, etc. these were things, the terminology that I was aware of, but I think it added a new, kind of, emphasis to things.”* (HCP18).

*Structuring the different news conversation:* We heard that the READY framework provided a structure for healthcare professionals to organise themselves; relate their existing and new knowledge to the experiences of parents and structure the different news conversations to address the communication elements parents were advocating for:

*“I’m much more careful with my words. I’m much more confident in doing things in a way… I just needed to first have like a structure. I think it’s always a stressful job to deliver different news but I’m much more confident and secure now.”* (HCP17)

On the SE-12 scale, the biggest difference in pre and post-training responses for participants was seen on Question 6: “How certain are you that you are able to successfully structure the conversation with the patient?” The mean score changed from 6.33 to 8.37 a difference of 2.04, 95%CI (1.75–2.33; p < 0.001) immediately after the training and 2.15, 95%CI (1.80–2.51; p < 0.001) a month post-training. This is an important finding which suggests that the READY framework potentially enabled healthcare professionals to have an evidenced-based format of how to structure and pace a different news conversation. To this end, the READY framework was also to be used by other interviewees to make structural changes across the organisation:

“*I think still, you know, a lot of people use the term bad news, which I think one of the things that we’ll be doing in our organisation is to slowly change that traditional approach.”* (HCP9).

### Variation in the impact of the training on knowledge

**Fig 3:**
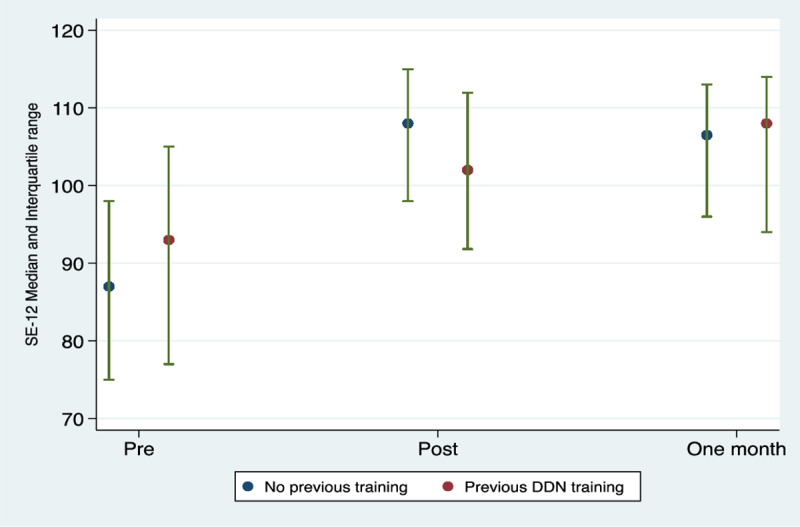
SE-12 Median and Interquartile range over time by prior delivering different news training.

Participants suggested that overall gain in skills and knowledge from the training was variable, with more experienced healthcare professionals potentially gaining less than newly qualified colleagues, as the former had experience in learning through practice and collaboration. This was also reflected in the quantitative data. Stepwise regression analysis to model the relationship between demographic/professional factors and post-training SE-12 (Fig 3) showed statistically significant differences between those who had previously received delivering different news training and those who hadn’t. Pre-training SE-12 scores were higher for the 40 participants who had received prior training in delivering different news, but the change from baseline was lower. The difference in the SE-12 change score between those who had received prior training and those who hadn’t was ‒7.36 (95% CI ‒12.5 to ‒2.25). The one-month post-training scores were similar for both groups.

### Beliefs about capabilities, skills, and knowledge

We examined the participants’ perceived capacity to deliver different news before training, their explanations for this and the perceived changes after the training. Two factors were central to these narratives: the lack of standardised training and the stress surrounding the delivery of different news.

#### Lack of standardised training.

Similar to our feasibility study, we found that central to interviewees’ beliefs about their capabilities, skills and knowledge was the fact there is no standardised training in delivering different news across the various professions that were represented in this study (5). 80.5% (n = 165) of the participants had not had any training in delivering different news. In our study, practitioners across all professions had developed their skill base through a combination of (unofficial) peer learning, personal experience and reading relevant materials as illustrated across obstetrics, sonography and midwifery:

*“I haven’t had any official training on stuff like this, it’s all just picked up as we go along throughout our training. So, it’s really nice to think there was something specifically designed to help us in this area because I think it’s something we all struggle with.”* (HCP10)

*“We’ve never been taught how to deliver, previously I would have said ‘bad news’ but now I say ‘different news’… (earlier in my career), we were just expected to go ahead and do it and I didn’t know whether I was doing it in the right way, the wrong way, was it acceptable to the patient…nobody fed back to me.”* (HCP8)

*“My experience was just having training on the job, so being on hand during scanning. Really just paraphrasing what colleagues had said, and therefore learning from them.”* (HCP4)

Interviewees acknowledged a wide range of levels of experience, understanding and ability in the peers and senior professionals they worked with, with implications for the modelling of good practice.

#### Stress related to the process of delivering different news.

Despite their experience, delivering different news remained stressful for many practitioners often due to an appreciation of the fact that parents vary so widely in their responses and that sometimes things did not go well despite good preparation:

*“Even now, I still have to do that to calm myself down and think about how I’m going to say that and sometimes I write things down to help me because it’s about getting the right terminology, pace and tone – so I know that I still find it a challenge.”* (HCP13)

*“I still find it difficult and I’m still very aware that, you know, it’s not a routine… it’s never a case of ‘Oh well I can just say that’ because it doesn’t work that way, everyone’s different.”* (HCP2)

The quotes above indicate that delivering different news may still be a challenging task despite training and experience largely because of an innate desire to get things right for families. This may explain why there was very little change in the post-training responses to the question ‘**I find delivering different news stressful’,** as shown in Table 5 below:

**Table 5:** Pre and post-training scores on delivering different news-related stress.

I find delivering different news stressful	Time		
	Pre-training n (%)	Post-training n (%)	One month post-training n (%)
Strongly Disagree	1 (0.5)	4 (2.1)	0 (0.0)
Disagree	7 (3.7)	17 (9.1)	14 (12.8)
Neither Agree nor Disagree	50 (26.7)	38 (20.3)	31 (28.4)
Agree	102 (54.5)	104 (55.6)	52 (47.7)
Strongly Agree	27 (14.4)	24 (12.8)	12 (11.0)
Total	187	187	109
Wilcoxon signed rank test		0.616	*0.0033*

### Environmental context and resources

We found that family demographics and organisational culture were factors that could impact the effective delivery of different news or uptake of the training.

#### Families’ demographics.

Interviewees noted the broad range of families they worked with and the challenges they faced to ensure that different news messages were understandable and meaningful to all parents. Some participants indicated a need to enhance the skills and structures gained from the training to support parents more equitably if English was an additional language, if parents had additional needs or lower literacy levels:

*“Because of the language barriers, some people would just say, there’s no heartbeat but they wouldn’t understand and after saying ‘your baby has died’ then they understand but it’s not the way you wanted to deliver it.*” (HCP5)

*“Especially if English isn’t the first language as well, that’s another challenge… and again, you know you’re then having to have your interpreter over the phone*.” (HCP7)

#### Organisational culture.

Participants suggested that the organisational culture could affect the uptake of delivering different news training as well as the effective delivery of different news. In some NHS trusts, participants had more flexibility to attend training due to their professional roles which had allocated training time. Financial resources to backfill staff who attended the training were variable which affected uptake of training. Furthermore, workload and work pressures meant some healthcare professionals would be unable to attend training as they would either need to do this in their own time or make up lost time/caseloads (which were already at capacity) on their return to work.

The organisational culture marked by persistent service pressures such as time, capacity and room availability was identified as a factor that could also be skills that healthcare professionals had developed for delivering different news:

“*I think anywhere you have a clinic booked, in that, you can’t step out, you can’t step away from, you know if you are the only person that can carry on with that scan list, you give the news, then it’s almost ‘goodbye, my colleague will sort you out’. It’s just, it’s unsatisfactory*.” (HCP3).

Some suggested that a broader culture change was needed to bring about a more conducive environment for delivering different news, rather than focusing on individual-level change for staff who did not deliver news very well due to pressures on staff. Similarly, respondents said that appropriate spaces for delivering different news were not always available, meaning that different news was often shared in environments that were not supportive to the parents. Whole system changes would be needed beyond the training to ensure that the provision of space to deliver different news is a priority as per the READY framework used in the training.

### Behavioural regulation

The data indicated that the training regulated healthcare professional behaviour in three ways: involving parents in the delivery of different news process more actively, providing balanced information and healthcare professional self-care.

#### Involving and empowering parents.

Interviewees described how they wanted to involve parents in various ways including being clear about the purpose of appointments and providing continuity of care wherever possible:

*“I think (couples) think it’s more of a social visit sometimes rather than a diagnostic view of their baby, so we have to sort of prepare them but nicely… so I’ve introduced that into my spiel because I feel that sort of sets them up to think, oh yes, you know, we’re not just here to find out the sex of our baby… so I think from that point of view they’re very attentive… I think it’s had a dual purpose of engaging everybody in why they’re here for the scan so that’s really helped.” (HCP4).*

The above experiences were also reflected in the quantitative findings. There was a statistically significant increase of 1.87 points (95% CI 1.59‒2.16; p < 0.001) in the mean score in response to Q2 “How certain are you that you are able to successfully make an agenda/plan for the conversation with the patient?” on the SE-12 Scale (the maximum score for each individual SE-12 question is 10). A lower but statistically significant increase was observed at one month 1.55 points increase (95% CI 1.21‒1.90; p < 0.001). There was also a statistically significant increase of 1.74 (95% CI 1.47–2.01; p < 0.001) in the mean score in response to Q3 on the SE-12 score: “How certain are you that you are able to successfully urge the patient to expand his or her problem/worries?”. These changes suggest that the training gave healthcare professionals the skills and the knowledge to actively seek to engage the families in the decision-making process and the care plan. This is an important aspect of ensuring patient agency and autonomy.

#### Optimism and balance.

During the training, the parent contributors reiterated how families often retained such a strong image of how they received their different news as a film played in their minds long after they had received it. One healthcare professional described how they are ensuring that they provided parents with more balanced information, or simply took time to focus on the baby or babies, noting this was a shift from how they would have previously presented information when the prognosis was poor. This is an important aspect of delivering different news which was highlighted by parents during the co-production process:

*“If we’d have found out that she had the heart condition and didn’t have Downs Syndrome nobody was going to sit down with us and say, you know, your baby’s got to have open-heart surgery when they’re born and, you know, do you want to put a baby through that, you know, do you think you might want to think about, you know, and there just wouldn’t have been that discussion. But all of a sudden because [name] has Downs Syndrome there’s, it’s changed it in some way and it’s, she’s almost less of a person, less of a child.”* (Co-production Parent 7)

We heard similar descriptions of more balanced and family-focused conversations from other practitioners, motivated by narratives from families with lived experience and a focus on building the parent-baby relationship where possible.

#### Self-care.

The training highlighted the emotional impact of delivering different news and the value of formalised supervisor support as well as informal peer support structures. There was a statistically significant increase in the scores for the importance of debriefing and accessing support from colleagues immediately after the training but not one month after the training as shown in Table 6 below:

**Table 6:** Pre and post-training scores on the importance of debriefing and accessing support.

It is important for me to be able to debrief and get support from colleagues	Time		
	Pre-training n (%)	Post-training n (%)	One month post-training n (%)
Strongly Disagree	0 (0.0)	2 (1.1)	0 (0.0)
Disagree	0 (0.0)	0 (0.0)	1 (0.9)
Neither Agree nor Disagree	7 (3.7)	3 (1.6)	0 (0.0)
Agree	76 (40.6)	51 (27.3)	40 (36.7)
Strongly Agree	104 (55.6)	131 (70.0)	68 (62.4)
Total	187	187	109
Wilcoxon signed rank test		*<0.001*	0.105

This was also reflected in the qualitative data

*“My approach is holding an awful lot in, and I do need to start talking to other people and getting that sort of support that is available in the team… it made me think about the emotional toll that it can have on you* (HCP1)

### Reinforcement

Here we the incentives that could increase the impact and sustainability of the delivering different news training.

#### Organisational and team culture.

**Fig 4:**
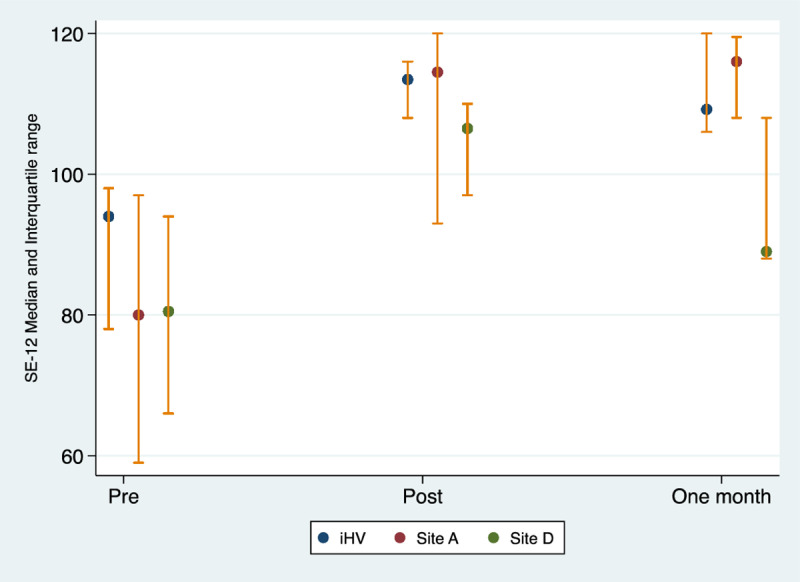
SE-12 Median and Interquartile range over time by Trainers.

Without mandatory delivering different newstraining, working towards a team culture around delivering different news can provide a strong ‘safety net’ which strengthens team capacity and addresses areas of concern. Our quantitative findings suggest that the cascade model of training is both feasible and acceptable. The percentage of delivering different news Champions who delivered one training to 15 or more healthcare professionals (non-Champions) was 60% (n = 12). Stepwise regression analysis showed statistically significant differences in SE-12 for participants who were trained by the research team (delivering different news Champions) and non-Champions trained in site A and site D but this was not seen for the other sites (see Fig 4). Pre-training SE-12 scores were lower for non-Champions trained at Site A and Site D, but post-training scores were similar to the delivering different news Champions who were trained by the research team. This is an important finding which suggests that with sufficient support, practice and training Champions may produce the same results as the research team. This can make delivering different news training sustainable in the long term. However, this finding must be interpreted with caution due to the small sample size and further research is needed to establish these findings.

#### Promoting the READY framework more widely.

While the READY framework was initially focused on different news regarding a congenital variation associated with a learning disability, the feedback from healthcare professionals has confirmed that it is more widely applicable and in fact, should be used for a greater range of scenarios to build a greater level of consistency in different news delivery across teams:

*“I had a patient with a new diagnosis of a probably oncological malignancy, they are 12, so not at all within the age range of the course, but… I was just really thinking… about the way I sat with the mother because it’s Covid-19 so we can only have one parent down, trying to speak to the dad on speakerphone, so just trying, so things like that making, you know, being mindful about thinking about my choice of words, how I go into things, how I lead into things… I was very conscious of wanting to allow both parents to be involved in the discussion, which I think definitely came across from the course, the importance of not just giving information with one, I think mainly the mother and then there are scenarios on the course, and, you know, giving the mother the burden of then having to explain that to her partner or her, you know, the rest of her support mechanism... So, I think, again, that was something that was really emphasised in the course, is being available to go back and have subsequent discussions.” (HCP18)*

Discussing whether the training would need a high degree of adaptation to be relevant to these broader ranges of situations, this manager felt that the core should remain, with the opportunity to broaden the discussion through the case studies used during the training:

*“I think, I think it is really relevant… that the training, not change it or adapt, but just to sort of make it relevant for other conversations, so really difficult conversations that you’ve got to have with that parent, and it could be down to even hygiene, the home environment, it could even be around things like obesity and diet. It really can morph itself into a lot of other situations that is, I think that’s where we will take our sustainability.”* (Manager 3)

Providing the training across various professions was considered essential as parent interviews emphasised the fact that delivering different news is a ‘process, not an event’ which involves a range of practitioners with different roles delivering a package of care:

*“A diagnosis isn’t a discrete event, there can be months and even years of getting little bits of information, so there was say a point where I knew that he was delayed but I didn’t know it was a genetic condition... when I first went into the conversation with the ophthalmologist I didn’t know, so when he said to me, when did he roll, when did he do this, when did he do that, he concluded he was delayed and that was a bit of a diagnosis, so there’s been multiple happenings like that along the journey where lots of different people have given us bits of information, said not always that sensitively.”* (Co-production Parent 5)

## Discussion

In this section we discuss the key findings for each research objective, the study limitations and key recommendations for research, policy and practice. This study had the following objectives:

- To update the delivering different news training intervention based on the recommendations from the feasibility study.
- To conduct a pre and post-test study to measure the impact of the training on the self-efficacy, knowledge, attitudes and practices of healthcare professionals who receive the delivering different newstraining.
- To conduct an in-depth qualitative exploration of the barriers and facilitators to the implementation of the training in practice from the perspective of healthcare professionals and managers.
- To assess the sustainability and scalability of providing the delivering different news training through a cascade model of training.

## Key findings

### Updating the delivering different news training intervention

We used purposive sampling to recruit parents with the lived experience of receiving different news about medical conditions which were not captured in the case studies for the training delivered during the feasibility study [33] .We were able to obtain six case studies of genetic medical conditions for which parents had received different news in the first 1001 days. These case studies consolidated the initial training supporting the relevance of the training to a much wider group than the initial training. However, participants indicated the need to further diversify the focus of the delivering different news training case studies to reflect a wider range of conditions or circumstances given that the READY framework could be widely applicable. This would potentially increase the training’s relevance to various other healthcare professionals and better support them in the reality of their role.

The delivering different news training and the READY Framework were initially developed to support the delivery of different news about a child having a condition associated with a learning disability during pregnancy or at birth using the Medical Research Council guidelines for the development and evaluation of complex interventions [5,27]. Following a feasibility study, the training has been expanded to the first 1000 days of a child’s life in the current study. Although we have not tested this specifically in this study, the qualitative data suggests that the READY framework might be useful outside of this setting. Several other frameworks may be used to communicate different news such as the SPIKE protocol (setting, perception, invitation, knowledge, empathise) and the ABCDE (Advance preparation, Build a therapeutic relationship, Communicate well, Deal with patient and family reactions, Encourage and validate emotions) model [38,39]. The main difference between the READY Framework and other frameworks such as the SPIKE protocol, is that the research team used the Medical Research Council guidelines for the development and evaluation of complex interventions to develop the READY Framework and the accompanying training. In addition, the training is underpinned by findings from the lived experiences of families who receive different news in the first 1000 days, healthcare professionals who deliver different news in that period; an extensive literature review and the use of relevant theory to address the barriers to implementation and aid diffusion of the training into everyday clinical practice [5,6,26].

If future training teams diversify cases, there is a need to support maintaining fidelity to the intervention by having a range of online content (with user ideas/guidance) that can be used flexibly.

### Measurement of the impact of the training on the self-efficacy, knowledge, attitudes and practices of healthcare professionals who receive the delivering different news training

The delivering different news training has the potential to improve self-reported confidence to deliver different news as well as healthcare professionals’ self-efficacy to deliver different news. There were statistically significant differences between pre- and post-training SE-12 scores at both post-training time points. SE-12 was significantly higher post-training. The estimated difference in mean scores post-training was 18.3 (95% confidence interval 15.7 to 20.9 p < 0.001), and one-month post-training 16.9 (95% confidence interval 13.7 to 20.2; p < 0.001), more than three times higher than the minimum important difference in SE-12 on which our sample size was based. Other studies have also reported that communication skills training has the potential to improve healthcare professionals’ self-efficacy as well as healthcare professional-families communication [22,23]. We examined the quantitative data to understand if these changes were the same across the six sites. There were no statistically significant differences in the change from baseline in SE-12 between trusts, although overall SE-12 scores for Site E were higher compared to the other trusts.

Among other things, the training gave healthcare professionals confidence and skills in the following areas: pacing the provision of information to the needs of the family, the use of the right language when delivering different news as well as provision of a structure for delivering the different news conversation using the READY framework. These are very important aspects of the delivering different news process which have been documented in similar studies [15,40,41]. These positive changes were also reflected in the quantitative data with the biggest difference in mean scores for the pre and post-training responses being on healthcare professionals’ ability to successfully structure the different news conversation with the patient. We also found that healthcare professionals were able to adapt the READY Framework beyond congenital variations in the first 1000 days for example in oncology or community settings where it can support difficult public health or safeguarding conversations with families. Indeed, other researchers from Australia indicated that the READY framework could be used as a communication skills training intervention in critical care [42]. In these additional settings, the wording in the setting would need to change from parent/families to patient, client or service user as deemed appropriate.

### Assessment of the sustainability and scalability of providing the delivering different news training through a cascade model of training

To assess the sustainability and scalability of providing the delivering different news training through a cascade model of training. We compared the observed changes in Self-Efficacy in healthcare professionals trained by the research team (Champions) and those trained by the Champions (non-Champions). We found a statistically significant difference between delivering different news Champions and non-Champions, SE-12 scores were generally higher for Champions and their improvement from pre-training was greater.

Although the changes observed in the non-Champions were different from the Champions, the results are promising as there was still a statistically significant increase in means scores although this was less than the changes observed in the Champions. It is important to bear in mind the challenging backdrop against which DDN Champions delivered their cascade training, particularly the COVID-19 pandemic. Delivering different news Champions delivered the training amidst the related pressures of social distancing measures, staff redeployment, staff members shielding, self-isolating, cancellation of non-essential training; staff dealing with the secondary impact of the pandemic as well as the impact of the pandemic on staff emotional and mental wellbeing as reported elsewhere [43–45]. In most NHS trusts, research was either completely stopped to focus on clinical care or research was limited to COVID-19 studies which had significant implications for the setup and conduct of non-Covid-19 related studies such as the DDN study [45]. Given this backdrop, we think it was a huge success that 5 out of the 6 sites were able to deliver the different news study as planned and produce the positive results that were observed.

To build the capacity of delivering different news Champions it would be important for them to continue to have opportunities to deliver the training so that they build their confidence and skills in delivering the training to enable them to have the same level of proficiency as the research team who have the advantage of several years of delivering related training to healthcare professionals.

### Barriers and facilitators to the implementation of the training in practice from the perspective of healthcare professionals and managers

Our analysis showed that delivering different news well was of fundamental importance to the interviewees, however, 80.5% (n = 165) of the participants had not had any training in different news delivery. This is similar to what we found in our feasibility study and has also been noted in other studies [5,15,46]. Practitioners across all professions had developed their skill base through a combination of (unofficial) peer learning, personal experience and reading academic or other relevant articles. There was a strong desire among the interviewees to improve services, reduce complaints from families, improve families’ experiences and help them understand and manage diagnoses. These were key facilitators in undertaking the training and implementing it in practice.

Organisational culture was identified as a key barrier to the uptake of delivering different news training as well as the effective delivery of different news. In some NHS trusts, participants had more flexibility to attend training due to their professional roles which had allocated training time. Financial resources to backfill staff who attended the training were variable which affected uptake of training. Furthermore, workload and work pressures meant some healthcare professionals would be unable to attend training as they would either need to do this in their own time or make up lost time/caseloads (which were already at capacity) on their return to work. Our feedback showed that practitioners overwhelmingly want to deliver different news well. However, they referred to barriers such as persistent constraints which influence how well different news is delivered in their units. This included lack of capacity, time, space, and stringent shift patterns which were identified as barriers that needed to be addressed alongside providing training to staff encouraging individual-level change to develop a more accommodating environment both for parents and for practitioners.

### Study Limitations

This study was well-designed to answer the research questions. The mixed-methods approach was useful for enabling the triangulation of quantitative data with qualitative data from healthcare professionals, parents, and managers. The use of a mixed-methods approach meant that the limitations inherent in qualitative and quantitative studies were negated by the triangulation of data from different sources.

Despite these strengths of the research methods, the study has some limitations. Firstly, the study was originally funded on condition that it would be completed in 12 months which made it challenging to conduct a randomised control trial to measure the effectiveness of the training intervention. We, therefore, opted to conduct a pre-post study to evaluate the training intervention. This design has been used extensively to evaluate communication skills training interventions for healthcare professionals [47–50]. Further research is required to understand the impact of delivering this type of training on the emotional and mental well-being outcomes of families who have news delivered to them by trained healthcare professionals. Ideally, this would need to be answered through the use of a randomised control trial.

Secondly, 18 healthcare professionals completed the pretraining survey either during or after the delivering different news training. Their pre-training outcomes were excluded from the statistical analysis. Despite several reminders, 89 participants did not complete the one1-month post-training follow-up survey. The results from both post-training surveys have been presented but should be interpreted bearing in mind that the groups may differ in important characteristics.

Thirdly, we struggled to recruit parents for post-training interviews. Our initial methodology was to ask delivering different news Champions to share a parent invitation letter and an information leaflet with parents to whom they had delivered different news about a child having a condition associated with a learning disability. We later received ethical approval to broaden this cohort to parents receiving any kind of different news about their baby. Delivering different news Champions did not manage to share many of these letters, often stating that it was not appropriate at the time. We did not hear back from any parents directly through this method. We only managed to recruit one parent using this method.

### Recommendations

The findings suggest that delivering different news training can improve the knowledge, skills, confidence and self-efficacy to deliver different news. Implementation of the training coupled with policy and structural level changes that support the effective delivery of different news may improve outcomes for families. We would recommend that the training be rolled out nationally and internationally. The applicability of the READY framework in Australia [42] outside of the first 1000 days is encouraging and suggests that this training could have a significant impact on healthcare professional communication skills in the UK and beyond. Going forward it would be imperative for Health Education England now NHS England to fund the training and development of delivering different news Champions with regional forums to provide support for Champions to deliver the training locally. The policy would need to be supportive for staff to be able to implement the training in practice. Further research is required to evaluate the impact of this training on families, staff and costs to the NHS health and social care.

## Conclusion

The delivering different news training aims to equip healthcare professionals to demonstrate empathy; show compassion; be flexible with the demands of their work; utilise kind, simple and truthful language; offer sufficient time to answer questions; and know when and where to refer families for further care and support. The significant improvements in confidence and skills reported by healthcare professionals suggest that the training may be effective in equipping healthcare professionals to deliver different news sensitively and compassionately. This represents a key aspect of the potential for the prevention of mental ill-health across the life course. It is crucial that this training is rolled out nationally to ensure that families who receive different news receive the timely, high-quality, compassionate personalised care and support they need when it matters the most.

## Notes

1 In this study, ‘congenital variation’ was the preferred term by our parent representative. It refers to genetic status that results in a child’s development being different from what is considered medically normal.
